# Biomechanical tactics of chiral growth in emergent aquatic macrophytes

**DOI:** 10.1038/srep12610

**Published:** 2015-07-29

**Authors:** Zi-Long Zhao, Hong-Ping Zhao, Bing-Wei Li, Ben-Dian Nie, Xi-Qiao Feng, Huajian Gao

**Affiliations:** 1 AML & CAMM, Department of Engineering Mechanics, Tsinghua University, Beijing 100084, China; 2 Center for Nano and Micro Mechanics, Tsinghua University, Beijing 100084, China; 3 School of Engineering, Brown University, Providence, RI 02912, USA

## Abstract

Through natural selection, many plant organs have evolved optimal morphologies at different length scales. However, the biomechanical strategies for different plant species to optimize their organ structures remain unclear. Here, we investigate several species of aquatic macrophytes living in the same natural environment but adopting distinctly different twisting chiral morphologies. To reveal the principle of chiral growth in these plants, we performed systematic observations and measurements of morphologies, multiscale structures, and mechanical properties of their slender emergent stalks or leaves. Theoretical modeling of pre-twisted beams in bending and buckling indicates that the different growth tactics of the plants can be strongly correlated with their biomechanical functions. It is shown that the twisting chirality of aquatic macrophytes can significantly improve their survivability against failure under both internal and external loads. The theoretical predictions for different chiral configurations are in excellent agreement with experimental measurements.

Through the long history of natural evolution, many plants have adopted optimal morphologies with enhanced physical properties at different structural levels. Chiral growth is prevalent in many plant organs, e.g. tendrils of towel gourds^1^ and cucumbers^2^, grapevines^3^, stems of petunias^4^, roots of wild-type Medicago truncatulas^5^, and chiral arrays of seeds on sunflower heads^6^. The chiral morphologies not only help the plant organs with their biological functions, e.g. supporting, climbing, anchoring, efficient packaging and photosynthesis, but can also contribute to their mechanical properties^7^. For example, the spiral grains of trees protect their stems against torsion-induced breakage in heavy wind^8^. In bamboo, multilayered and helically-wound bast fibers greatly improve the deformability and strength of the plant^9^,^10^. The twisting morphology of *Paphiopedilum dianthum* flower petals can help release the residual stress induced by differential growth^11^,^12^.

Of particular interest to our present study is a class of plants called emergent aquatic macrophytes which possess twisted chiral morphologies. Emergent aquatic plants commonly live in the shallow water regions of lakes, ponds, and wetlands. In order to accommodate water-level fluctuations, they usually need to have erect, sufficiently long emergent organs (e.g. stalks and leaves) with reduced cross-sectional areas for various biological functions (e.g. photosynthesis, air delivery to roots) and survival in their living environment. For the slender emergent plants to withstand wind loads, twisted chiral structures would offer a substantial advantage. For example, twisted leaves in bulbous plants, bur-reeds, and grasses not only offer greater resistance to flexion, but also reduce wind drag with their chiral configurations^13^. Recently, it was reported that twisted chiral morphology can help a vertical leaf to achieve a greater height, without loss in structural stability^14^.

In this study, we explore a variety of representative aquatic macrophytes with twisted chiral morphologies, including *Scirpus rosthornii* Diels, *Sagittaria trifolia* L., *Schoenoplectus tabernaemontani* (C. C. Gmel.) Palla, *Acorus calamus* L., *Sparganium stoloniferum* (Graebn.) Buch-Ham. Ex Juz, and *Typha orientalis* C. Presl. It is observed that these slender plants, albeit living in the same natural environment, exhibit greatly different morphologies. For instance, the leaves of *Typha orientalis* display prominent twisting configurations which were described by Leonardo da Vinci over five centuries ago^15^. However, hardly any chirality is observed in the culms of *Schoenoplectus tabernaemontani*. To probe if there is a mechanical principle of chiral growth in aquatic macrophytes, we performed systematic measurements of the morphologies, multiscale structures, and mechanical properties of the emergent stalks/leaves of the six typical emergent aquatic plants. A biomechanical model is presented to clarify the chiral growth tactics adopted by these plants. It is shown that the twisting chirality can greatly improve both the axial stability and transverse bending resistance of the slender emergent stalks/leaves. Our theoretical predictions for the chiral morphologies of different plants exhibit good agreement with experimental measurements, demonstrating the crucial role of chirality in improving the survivability of plants under both internal and external loads.

## Results

### Morphology

**Figure 1:**
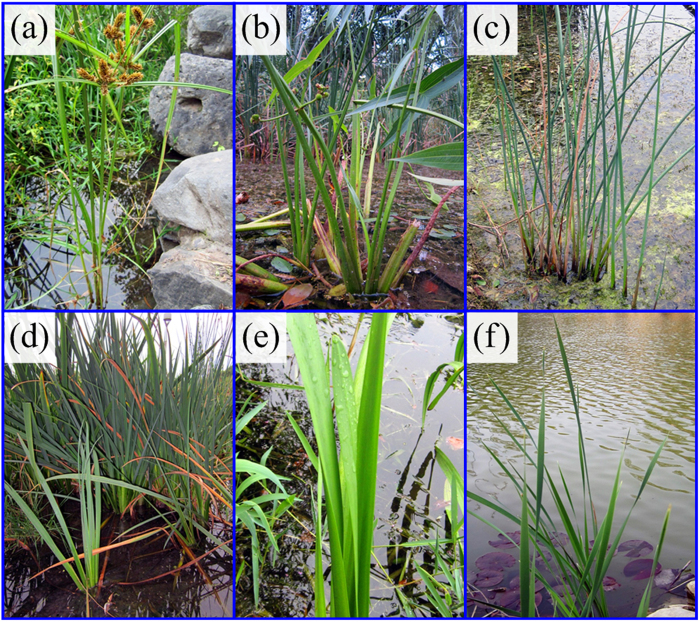
Emergent aquatic macrophytes. (**a**) *Scirpus rosthornii*, (**b**) *Sagittaria trifolia*, (**c**) *Schoenoplectus tabernaemontani*, (**d**) *Acorus calamus*, (**e**) *Sparganium stoloniferum*, and (**f**) *Typha orientalis*. All these photographs were taken by the first author Zi-Long Zhao.

We collected the emergent parts of six representative aquatic plants in the same freshwater habitat, including (a) the culms of *Scirpus rosthornii* with spikelets and leaves, (b) the petioles of *Sagittaria trifolia* with sagittate or somewhat hastate leaves on their heads, (c) the culms of *Schoenoplectus tabernaemontani* with spikelets, (d) the leaves of *Acorus calamus*, (e) the stems and leaves of *Sparganium stoloniferum*, and (f) the stems and leaves of *Typha orientalis* (Fig. 1). These plants are categorized into two groups, I and II, according to whether their slender emergent structures support any head organs or not on the upper end. Group I includes *Scirpus rosthornii*, *Sagittaria trifolia*, and *Schoenoplectus tabernaemontani* (the first row in Fig. 1), which are subjected to a compressive force due to the weight of their head organs (spikelets and/or leaves); while Group II (the second row in Fig. 1) includes *Acorus calamus*, *Sparganium stoloniferum*, and *Typha orientalis*, which are freestanding at the upper end. In what follows, we will show that the two categories are distinctly different both in overall chiral morphology and cross-sectional geometry, indicating that different strategies have been evolved to efficiently bear external (e.g., wind) and internal (weight) loads.

**Figure 2:**
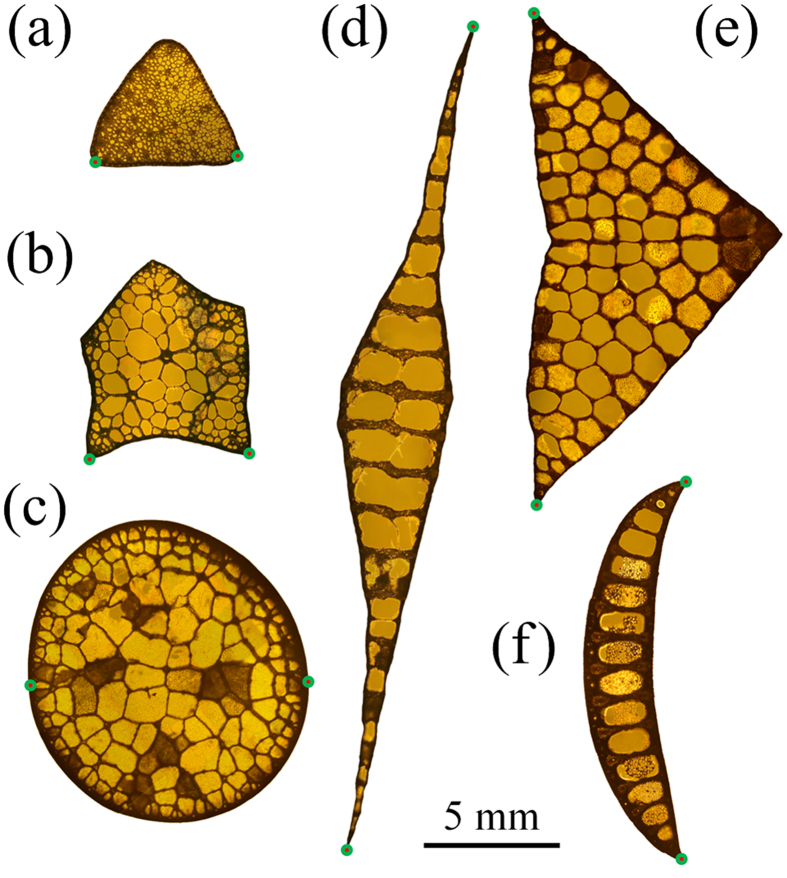
Cross sections of six species of aquatic macrophytes and their inner microstructures. (**a**) *Scirpus rosthornii*, (**b**) *Sagittaria trifolia*, (**c**) *Schoenoplectus tabernaemontani*, (**d**) *Acorus calamus*, (**e**) *Sparganium stoloniferum*, and (**f**) *Typha orientalis*.

Optical microscope observations of typical cross sections of the emergent stalks/leaves of the six aquatic macrophytes show unique parenchyma foam microstructures with well-developed air delivery channels (Fig. 2). It is interesting to find that these plants have different cross sections, with the shape close to an equilateral triangle for *Scirpus rosthornii* (Fig. 2a), pentagon for *Sagittaria trifolia* (Fig. 2b), circle for *Schoenoplectus tabernaemontani* (Fig. 2c), narrow-belt for *Acorus calamus* (Fig. 2d), isosceles triangle for *Sparganium stoloniferum* (Fig. 2e), and crescent moon for *Typha orientalis* (Fig. 2f). In addition, the cross sections of all six emergent aquatic plants are featured by porous structures, with denser parenchyma near the outer surfaces, especially in the vicinity of corners and epidermis regions, than that in the central regions.

**Figure 3:**
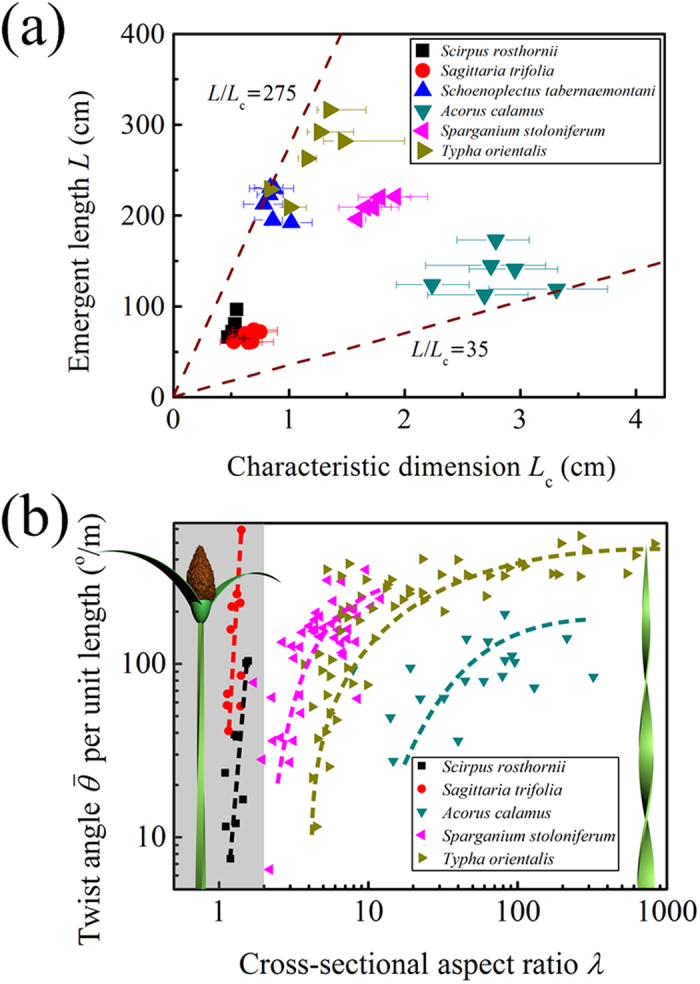
(**a**) Variation of the emergent length *L* versus cross-sectional characteristic size *L*_c_ of the six species of aquatic macrophytes under study. (**b**) Twist angle 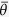 per unit length versus the cross-sectional aspect ratio *λ* of five aquatic macrophytes (not including *Schoenoplectus tabernaemontani*). The plants in Fig. 3b were drawn by the first author Zi-Long Zhao.

Next we measured the morphologies of the emergent stalks/leaves, including their cross-sectional sizes, slenderness ratios, and twist angles. For each aquatic macrophyte, a sufficiently large number of samples were measured. For the measured stalks/leaves of *Scirpus rosthornii*, *Sagittaria trifolia*, *Schoenoplectus tabernaemontani*, *Acorus calamus*, *Sparganium stoloniferum*, and *Typha orientalis*, the average lengths *L* are in the ranges of 80.7 ± 15.7 cm, 67.3 ± 6.8 cm, 211.3 ± 19.3 cm, 142.8 ± 30.3 cm, 208.5 ± 12.5 cm, and 262.8 ± 53.8 cm, respectively. The average twist angles *θ* of the measured stalks/leaves are 29.2° ± 17.1° for *Scirpus rosthornii*, 183.7° ± 133.3° for *Sagittaria trifolia*, 92.7° ± 42.7° for *Acorus calamus*, 288.4° ± 76.1° for *Sparganium stoloniferum*, and 674.9° ± 160.0° for *Typha orientalis*. respectively. Two representative points (the green dots labeled in Fig. 2) were specified on the outer boundary of each cross section, and their distance, *L*_c_, was taken as a characteristic size. The slenderness of an emergent stalk/leaf corresponds to the ratio of its length *L* to cross-sectional size *L*_c_. The results for the emergent stalk/leaf lengths *L* and the cross-sectional characteristic sizes *L*_c_ are plotted in Fig. 3a, where each data point corresponds to one intact plant, and the error bars indicate the variations of *L*_c_ along the longitudinal direction. The slenderness ratios of all emergent aquatic macrophytes, except *Acorus calamus*, are larger than 100; *Schoenoplectus tabernaemontani* and *Typha orientalis* have the largest slenderness up to about 275.

In order to reveal the chiral growth tactics of aquatic macrophytes, we attempt to establish the relationship between the twisting chiral configurations of the emergent stalks/leaves and their cross-sectional geometry. The aspect ratio of the cross section, denoted as *λ* (*λ* ≥ 1), is defined as the ratio between its maximal and minimal principal moments of inertia. The twist angle 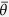 per unit length of the emergent structure is measured from the total twisting angle divided by length. The measured 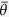 is shown as a function of *λ* in Fig. 3b for the emergent parts of aquatic macrophytes under investigation, except for the stalk of *Schoenoplectus tabernaemontani*, which has an approximately circular cross section with *λ* ≈ 1 and hardly grows in a chiral manner. From the base to apex, each stalk/leaf sample was cut along the axial direction into segments of about 20 cm in length. For the aquatic plants in Group I (*Scirpus rosthornii* and *Sagittaria trifolia*), the twist angle 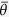 per unit length increases very rapidly and almost linearly with increasing *λ* in a logarithmic plot, while for the other three plants in Group II, 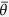 increases rapidly for smaller *λ* and saturates to a constant value with further increase in *λ*. For the emergent stalks of *Scirpus rosthornii* and *Sagittaria trifolia*, the cross-sectional aspect ratios *λ* vary in a narrow range from 1 to 2 but their twist angles 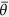 change from 10°/m to 600°/m.

### Mechanical properties

**Figure 4:**
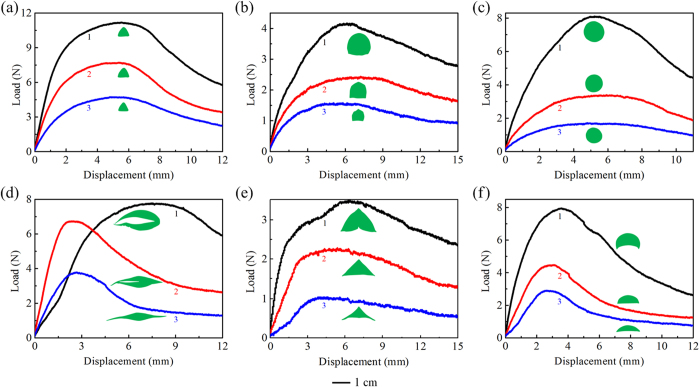
Typical load–displacement curves of six species of aquatic macrophytes. (**a**) *Scirpus rosthornii*, (**b**) *Sagittaria trifolia*, (**c**) *Schoenoplectus tabernaemontani*, (**d**) *Acorus calamus*, (**e**) *Sparganium stoloniferum*, and (**f**) *Typha orientalis*.

Three-point bending tests were then performed to measure the bending properties of the six species of aquatic macrophytes. To test their variations along the height direction, three typical load–displacement curves at different positions in each plant are plotted in Fig. 4, where the numbers 1–3 label the three positions from the base to the apex, respectively. The real cross-sectional geometry corresponding to each curve is also shown with the same scale bar beneath the plot. Among these plants, the stalk of *Scirpus rosthornii* (Fig. 4a), whose maximum load exceeds 10 N, is endowed with the greatest bending resistance, while the emergent stalks of *Sagittaria trifolia* (Fig. 4b) and leaves of *Sparganium stoloniferum* (Fig. 4e), whose maximum loads are about 4 N, exhibit the lowest resistance. The peak loads of the other three plants are moderate, in the range of about 7.8–8.1 N. The bending stiffness and strength of all plants show a decreasing tendency from the base to the apex.

**Figure 5:**
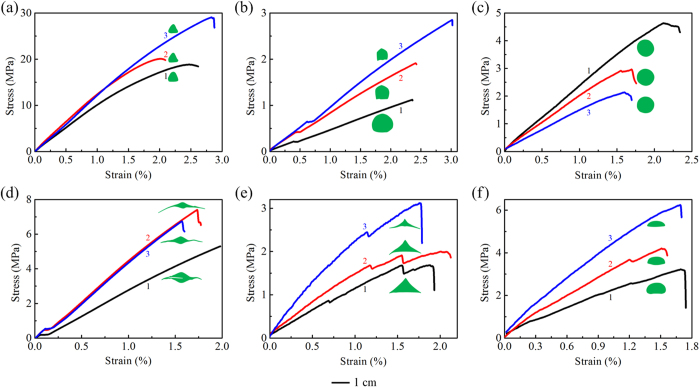
Typical stress–strain curves of the emergent stalks/leaves of six species of aquatic macrophytes. (**a**) *Scirpus rosthornii*, **(b**) *Sagittaria trifolia*, (**c**) *Schoenoplectus tabernaemontani*, (**d**) *Acorus calamus*, (**e**) *Sparganium stoloniferum*, and (**f**) *Typha orientalis*.

Uniaxial tension tests were performed to determine the elastic modulus of the emergent stalks/leaves, which will be used in our theoretical analysis. The stalks/leaves of intact plants were cut into a few segments and then used as specimens in the tests. For each plant, three typical stress–strain curves, as well as the corresponding real cross-sectional profiles, are presented in Fig. 5, where the numbers 1–3 label the measured positions from the base to the apex, respectively. The tensile stress on a cross section was calculated from the tensile force divided by the cross-sectional area. The elastic modulus of the material was determined from the linear regime of the stress–strain curves. The elastic modulus, except for that of *Schoenoplectus tabernaemontani* (Fig. 5c), shows an increasing tendency from the base to the apex. This can be understood by considering the relative porosity of the cross sections. According to our measurements, the porosity in the cross sections of a *Schoenoplectus tabernaemontani*’s culm remains almost constant along the height direction, but the same property increases for the other five aquatic macrophytes. Near the base of the stalk of *Scirpus rosthornii*, the elastic modulus is about 1 GPa (Fig. 5a), about one order of magnitude higher than that of *Sagittaria trifolia* (Fig. 5b). The elastic modulus of the other four plants changes in the range of 0.1–1.0 GPa.

In addition, it is noticed that the cross sections of the emergent stalks/leaves in the plants of Groups I and II exhibit different tendencies of variation along the height direction, as shown in Figs. 4 and 5. For the plants in Group I, both the shape and aspect ratio of the cross sections shrink slightly from the base to the apex such that the stalk can efficiently support the weight of the head organs. For the plants in Group II, However, the cross-sectional shape of the emergent leaves has a significant variation along the growth direction because it does not need to bear the weight of a head organ.

## Discussion

### Chiral morphologies of different aquatic macrophytes

A slender and erect emergent stalk/leaf is of crucial importance for aquatic macrophytes to achieve multiple biological functions, e.g. conducting photosynthesis and adapting to water-level fluctuations. A plant can optimize its structure from different aspects, e.g., hollow and functionally gradient stem structures, in order to reach a sufficient height (Fig. 2). Here we demonstrate, both experimentally and theoretically, that chiral growth is another efficient and robust strategy for the plants to reach a great height and, simultaneously, have an improved resistance against mechanical failure. For these aquatic macrophytes, the measured twist angles 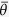 of the emergent stalks/leaves per unit length are shown in Fig. 3b as a function of their cross-sectional aspect ratios *λ*. It is clearly seen that the twisting chirality is prominent in most emergent stalks/leaves under study, with the twist angle up to several hundred degrees per meter in length. The six plants, albeit living in the same water region, show distinctly different 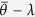 relations. The chiral morphologies of the emergent stalks/leaves are closely correlated with their slenderness and cross-sectional geometry. The 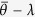 curves are very different for Group I (with head organs) and II (without head organs), as highlighted by the gray- and white-background colors in Fig. 3b, respectively. The stalks of *Schoenoplectus tabernaemontani*, with a nearly circular cross-section (*λ* ≈ 1), have negligible twisting chirality even though their slenderness ratio exceeds 200. This is because chiral morphology would not significantly affect the bending and buckling behaviors of an emergent stalk with circular cross section. The emergent stalks of *Scirpus rosthornii* and *Sagittaria trifolia* in Group I show an approximately linear 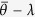 relation in the logarithmic plot. The 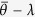 curves of plants in Group II, however, are nonlinear: 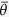 first increases rapidly with *λ* and then gradually approaches a constant. In Group II, the emergent leaves of *Acorus calamus* exhibit a more narrow cross-section, i.e. a larger cross-sectional aspect ratio *λ*, than those of *Sparganium stoloniferum*. These observations suggest a close correlation between the twisting morphology and cross-sectional geometry of the emergent stalks/leaves of aquatic macrophytes.

From the experimental observations, an interesting question arises: Why do aquatic macrophytes living in the same region adopt distinctly different tactics of chiral growth? Here we attempt to answer this question by considering the relations among geometry, mechanical properties, biological functions and environment of these plants. In the natural environment, aquatic macrophytes are subjected not only to internal loads due to the weight of their head organs and the self weight of stalks/leaves, but also to external loads resulting from the water flow, wind, rain, birds, etc. We hypothesize that the chiral growth of aquatic macrophytes is a result of an evolutionary strategy to enhance the mechanical properties needed to accomplish a variety of biological functions under different environmental conditions. For the aquatic macrophytes, chiral growth is an important tactics of structural optimization. In what follows, we develop a theoretical model to investigate how the aquatic plants strengthen themselves by resorting to chiral growth.

**Figure 6:**
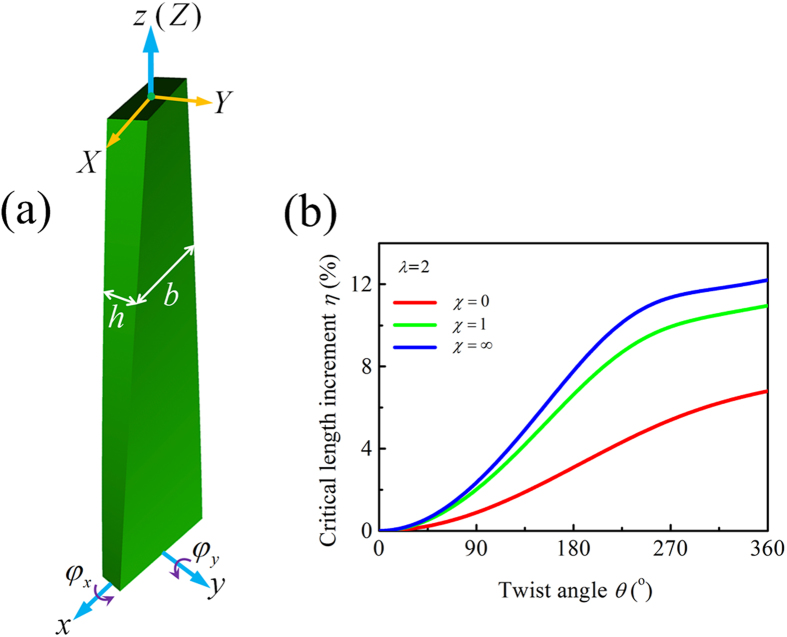
A pre-twisted cantilever beam model of the emergent stalks/leaves of aquatic macrophytes. (**a**) Schematic of the model and (**b**) increment *η* of the critical buckling length as a function of the twist angle *θ*.

In the model, the emergent stalk/leaf is regarded as a pre-twisted cantilever beam with a solid and rectangular cross-section, as shown in Fig. 6a. As to the boundary conditions, the beam is clamped at the bottom end and free at the top. Let *b*, *h*, and *L* denote its width, thickness, and length, respectively, with *b* ≥ h. The cross-sectional aspect ratio is expressed as *λ* = *I*_max_/*I*_min_ = *b*^2^/*h*^2^, where *I*_max_ and *I*_min_ denote the maximal and minimal principal moments of inertia of the cross-section, respectively. The aquatic macrophyte stalks/leaves with different *λ* values shown in Fig. 3b can be modeled by specifying different values of *b* or *h*. The material is assumed to be homogeneous, isotropic, and linear elastic. The beam has a twist angle 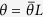 along its longitudinal direction. A Cartesian coordinate system (*x*, *y*, *z*) is attached, with origin *o* located at the cross-sectional centroid at the clamped end of the beam, and *x*, *y*, and *z* axes parallel to the width, thickness, and length directions, respectively.

### Twisting chiral morphology improves the stability against axial buckling

Our experiments showed that, though living in the same natural environment, the aquatic macrophytes in Groups I and II are drastically different in their cross-sectional geometry (Fig. 2) and twisting morphology (Fig. 3b). This suggests that they may have adopted different strategies of chiral growth to enhance their survivability. Here, we first consider the aquatic macrophytes in Group I, in which the stalks carry head organs (e.g., spikelets and leaves) atop their emergent stalks. The cross sections of these plants are featured by a small aspect ratio *λ*. Specifically, the cross sections of *Scirpus rosthornii* and *Sagittaria trifolia* assume, respectively, a triangular and a pentagonal shape with a smaller aspect ratio in the range of 1 < *λ* < 2, while the cross section of *Schoenoplectus tabernaemontani* is close to a circle with *λ* ≈ 1. The total weight of the head organs is comparable to that of the emergent stalk itself. For each kind of the plants in Group I, we measured the weight of 5 individuals, consisting of their stalks and head organs. The relative weights of the head organs with respect to the emergent stalk are 0.59 ± 0.26 for *Scirpus rosthornii* and 0.46 ± 0.10 for *Sagittaria trifolia*, respectively. Therefore, the stalks are subjected to not only their own weight but also the weight of head organs, and the latter plays a dominant role in the mechanical behavior of the stalks.

According to the theory of beams, Euler buckling is a typical failure mechanism for a free-clamped slender beam subjected to a concentrated compressive force at its upper end and a distributed load along its length direction. Therefore, we analyze the axial buckling behavior of a pre-twisted beam with rectangular cross section. Let *q* denote the distributed force (self-weight) per unit length and *P* the concentrated load (head organs) acting at the free end. The relative value of the two loads is defined as the dimensionless load ratio *χ*  =  *P*/(*qL*_ref_), *L*_ref_ being a reference length. Let the dimensionless parameter *η* = (*L*_cr_ − *L*_0_)/*L*_0_ quantify the enhancing effect of twisting chirality on the structural stability, where *L*_cr_ and *L*_0_ represent the critical buckling lengths of a pre-twisted beam and an untwisted counterpart, respectively. We have used the principle of minimum potential energy to estimate *η*^11^. The detailed derivation is given in the Supplementary Information.

According to our measurements of *Scirpus rosthornii* and *Sagittaria trifolia*, we took the following representative geometric and material parameters: width *b* = 5 mm, thickness 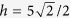 mm (*λ* = 2), Young’s modulus *E* = 1 GPa, Poisson’s ratio *ν* = 0.3, and reference length *L*_ref_ = 1 m. Three typical loading conditions were considered: (i) *P* = 0 and *q* > 0; (ii) *P* = *q* > 0; and (iii) *P* > 0 and *q* = 0, which correspond to the load ratio *χ* = 0, 1, and ∞, respectively.

The enhancing effect of chiral morphology on the critical buckling length is studied by plotting *η* as a function of the twist angle *θ*, as shown in Fig. 6b. For the pre-twisted beam, *η* is always positive and increases with *θ*. For a specified *θ*, the larger the load ratio *χ*, the higher the critical length *L*_cr_. When *θ* = 360°, for instance, *η* increases more than 60% as *χ* changes from 0 to 1. Therefore, the enhancing effect of chiral morphology is more significant for a plant stalk subjected to a concentrated load (the weight of head organs) than that under a distributed force. The twisting stalks of *Scirpus rosthornii* and *Sagittaria trifolia*, which carry head organs, have cross sections with a smaller *λ* such that they can effectively protect themselves from axial buckling in any directions. Figure 3b illustrates that the *λ* value of *Scirpus rosthornii* and *Sagittaria trifolia* varies in the range from 1 to 2. For these plants with *λ* > 1, chiral growth can improve the axial stability against Euler buckling. It is seen from Fig. 6b that the critical length *L*_cr_ increases by about 11% when *λ* = 2, *θ* = 360°, and *χ* = 1. The twisting morphology becomes more significant as *λ* increases, as shown by the 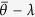 relations in Fig. 3b. Besides, twisting chirality has no effect on the axial buckling resistance when *λ* = 1. Thus, chiral growth hardly occurs in the emergent stalk of *Schoenoplectus tabernaemontani* with *λ* ≈ 1. It is also seen from the curve of *χ* = 0 in Fig. 6b that chiral growth can improve the ability of Group II plants against axial buckling, though they do not have head organs.

### Twisting chiral morphology improves the bending resistance against transverse deformation

Now we explore the biomechanical tactics of the aquatic macrophytes in Group II, which have no head organ. In contrast to the plants in Group I, the cross-sectional aspect ratio *λ* of the emergent leaves in Group II varies in a much wider range from 2 to 800. In addition, the cross sections of these structures become narrower and narrower along the height direction, as can be seen from the transections in Figs 4 and 5. Correspondingly, the cross-sectional aspect ratio *λ* of the emergent leaves increases from the base to the apex. For the three plants, because of the absence of concentrated compressive load exerted on the top, the bending deformation induced by wind is the main cause that may break the emergent leaves. For a straight beam with a narrow cross section, the deflection is anisotropic and depends on the direction of the distributed wind load because of the direction-dependent bending stiffness. The emergent leaf will exhibit a much larger deformation when the wind blows toward its wide faces than that in the case when the wind is parallel to the wide faces. Thus, a straight leaf without a twisting chiral morphology would be susceptible to breaking in the flexible direction. In this case, a chiral morphology could substantially improve the survivability of the plant, as demonstrated below.

To reveal the effect of chiral growth of aquatic plants under bending, we analyzed a pre-twisted beam with rectangular cross section subjected to a distributed transverse force ***f***(z). Denote the force intensity per unit area as 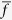 and assume that ***f***(z) is proportional to the windward area of the leaf. Let *γ* denote the direction of the wind measured from the *y*-axis. The derivation of the deflections *u*_*x*_(*z*) and *u*_*y*_(*z*), which are normalized as 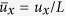 and 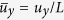, is given in the Supplementary Information. The normalized total deflection at the free end of the beam is 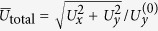, where *U*_*x*_ = *u*_*x*_(*L*), *U*_*y*_ = *u*_*y*_(*L*), and 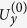 denotes the value of *U*_*y*_ when *θ* = 0° and *γ* = 0°. The maximum normal stress in the beam, *σ*_max_, is normalized as 
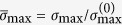
, where 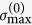 denotes the maximum normal stress in the case of *θ* = 0° and *γ* = 0°.

**Figure 7:**
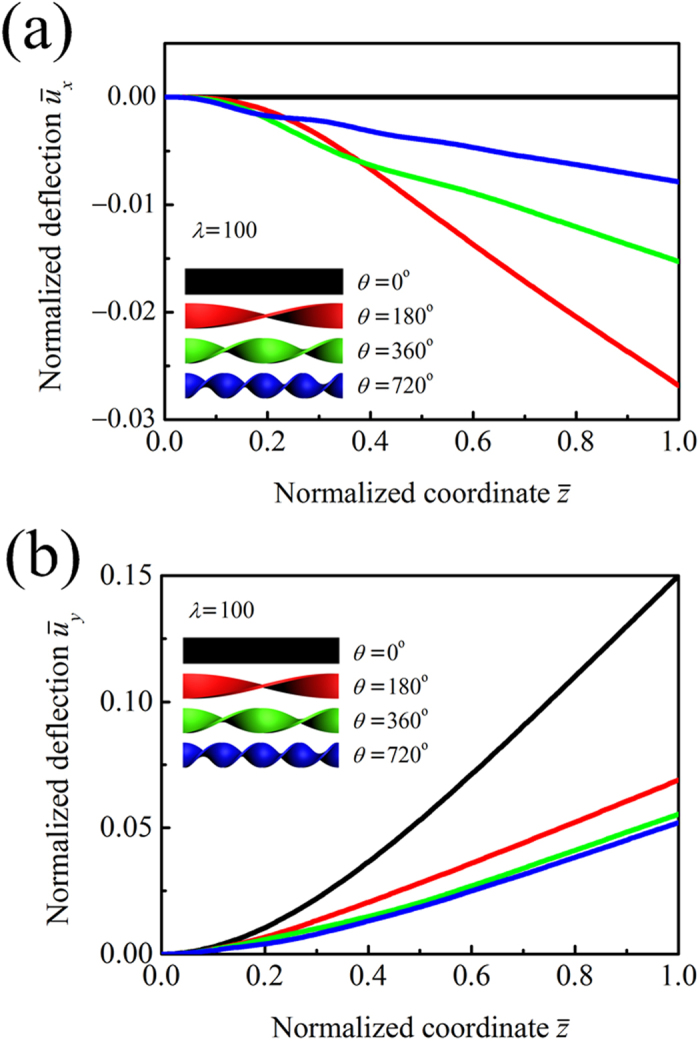
Variation of normalized deflections. (**a**) 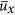 and (**b**) 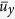 with respect to normalized coordinate 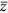.

Based on the experimental measurements, we take the following model parameters: beam length *L* = 1 m, beam width *b* = 1 cm, Young’s modulus *E* = 1 GPa, Poisson’s ratio *ν* = 0.3, and force intensity 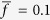 N/m^2^. The normalized deflections 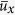 and 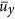 of the pre-twisted beam are plotted in Fig. 7 as a function of the normalized coordinate 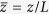, with the thickness of *h* = 1 mm (*λ* = 100), force direction of *γ* = 0°, and twist angles of *θ* = 0°, 180°, 360°, and 720°. When *θ* is small, the free-end deflection in the *x* direction is much smaller than that in the *y* direction. As can be seen from the curves of *θ* = 180°, 360°, and 720°, the deflection *U*_*y*_ of a pre-twisted beam is distinctly smaller than that of a straight beam (*θ* = 0°).

**Figure 8:**
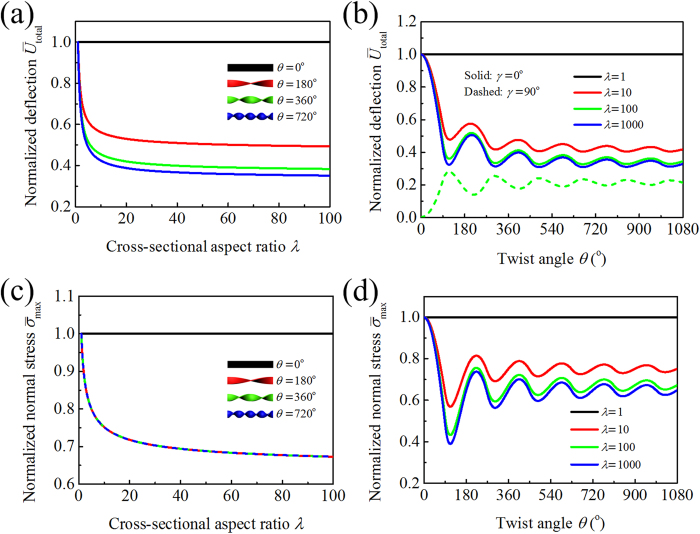
Normalized deflections 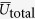 as functions of (**a**) the cross-sectional aspect ratio *λ* and (**b**) the twist angle *θ*. Normalized normal stress 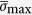 with respect to (**c**) the cross-sectional aspect ratio *λ* and (**d**) the twist angle *θ*.

Next we fixed the beam width *b* = 1 cm and changed the beam thickness *h* from 1 cm to 1 mm, corresponding to the increase of the aspect ratio *λ* from 1 to 100. The variation of the normalized free-end deflection 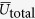 is plotted in Fig. 8a, where we take the force direction at *γ* = 0°, and twist angles at *θ* = 0°, 180°, 360°, and 720°. It is seen from the curves of *θ* = 180°, 360°, and 720° in Fig. 8a that the normalized deflection 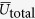 of the pre-twisted cantilever beam decreases significantly with *λ*. For example, when *θ* = 360°, 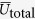 decreases more than 60% as *λ* changes from 1 to 100. Specifically, when the cross-sectional aspect ratio *λ* = 1, the normalized deflection is fixed at 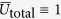, which does not vary with *θ*. Therefore, for emergent leaves or stalks with *λ* = 1 (e.g., those of *Schoenoplectus tabernaemontani*), a chiral morphology would not benefit the bending resistance.

The normalized deflection 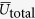 is plotted in Fig. 8b as a function of the twist angle *θ* from 0° to 1080°, where we took *γ* = 0° and several representative values of *λ*. Once the force intensity 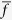 is specified, the normalized deflection 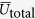 depends on both the structural stiffness and the windward area of the beam. As the twist angle *θ* increases, the bending stiffness of the beam is monotonically improved while the windward area may either increase or decrease. Therefore, the normalized deflection 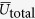 of the pre-twisted beams exhibits some oscillations with increasing *θ*. At first, 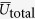 decreases rapidly, demonstrating a high sensitivity of the beam’s deflection to twist when *θ* is small. As *θ* increases, however, the variation of 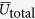 becomes slower. Thus one can easily understand the changing tendency of the twist angle 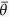 per unit length of *Acorus calamus*, *Sparganium stoloniferum*, and *Typha orientalis,* as shown in Fig. 3b. These results also provide a feasible explanation of why the measured 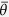 for the emergent leaves of the aquatic macrophytes in Group II is usually smaller than 600°/m, as further increase does not lead to more significant improvement in bending resistance.

It can be further seen from the curves in Fig. 8b that for a beam with *λ* > 1, twisting it into a chiral morphology can significantly improve its bending resistance. Figure 8b also shows that twisting chirality improves bending stiffness more significantly for beams with larger aspect ratios *λ*. Therefore, our model predicts that the twist angle 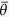 per unit length should increase with increasing *λ*. This theoretical prediction is consistent with our experimental observations on *Acorus calamus*, *Sparganium stoloniferum*, and *Typha orientalis* whose cross-sectional aspect ratios *λ* vary along the height direction.

We also examined the effect of twisting chiral morphology on the bending anisotropy of a pre-twisted beam with *λ* > 1. When the clamped-free beam in Fig. 6a is untwisted (*θ* = 0°), it has a wide face in the (*x*, *z*) plane and a narrow face in the (*y*, *z*) plane. We compared the deflections of a twisted beam when the wind load is perpendicular to either the (*x*, *z*) plane (*γ* = 0°) or the (*y*, *z*) plane (*γ* = 90°). The normalized total deflection 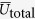 is plotted in Fig. 8b as a function of the twist angle *θ*. When *θ* = 0°, the two cases of *γ* = 0° and 90° exhibit the largest difference in 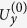. As *θ* increases, the difference of the two 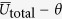 curves first decreases rapidly and then approaches a constant in an oscillatory manner. Therefore, twisting a beam into a chiral morphology can greatly reduce its anisotropy in transverse bending resistance. This property seems crucial for the emergent leaves of *Acorus calamus*, *Sparganium stoloniferum*, and *Typha orientalis*, which have a narrow cross section, to withstand wind load coming from different directions. Therefore, for the aquatic macrophytes in Group II, which do not have head organs, chiral growth is an important strategy to reduce transverse bending anisotropy. This mechanism greatly improves their ability to resist wind from any direction.

The variation of the normalized maximum normal stress 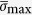 with respect to the cross-sectional aspect ratio *λ* is plotted in Fig. 8c, where we take the force direction at *γ* = 0° and the twist angle at *θ* = 0°, 180°, 360°, and 720°. It is seen that 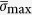 in the pre-twisted cantilever beam decreases significantly with the increase in *λ*. For example, when θ = 360°, 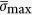 decreases more than 30% as *λ* changes from 1 to 100. For a beam with *λ* = 1, the maximum stress does not vary with *θ*. For a beam with *λ* > 1, twisting it into a chiral morphology will substantially decrease the maximum normal stress 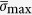 (Fig. 8d).

It is emphasized that the chiral morphology of the slender aquatic macrophytes under investigation may have a multitude of mechanical functions, e.g., reducing the maximum stress and deformation, promoting reconfiguration, and improving the resistance of lodging and axial buckling. These mechanisms all help the plants to better adapt to different environmental conditions. Our analysis here shows that the chiral morphology can greatly reduce the transverse anisotropy of bending deformation as well as the maximum normal stress in the plants. Due to the chiral growth tactics, the plant will have almost identical deformations no matter what directions the wind comes from. For the emergent plants, this mechanism might effectively improve the lodging resistance of their stalks/leaves.

It is also worth mentioning that we have not accounted for some other possible mechanisms associated with the chiral morphology, e.g., wind-induced torsion, wind-adaptive reconfiguration, and flutter instability. Due to the wind-induced torque, the narrow cross section of the stalk/leaf would tend to align with the air flow. The aerodynamic effects should be further investigated through fluid dynamics simulations and wind tunnel experiments. Furthermore, to accommodate wind loads, the stems and leaves of plants generally reconfigure themselves by, e.g., streamlining^16^,^17^, flapping^18^, bending and twisting^19^. The wind-adaptive reconfiguration is an essential mechanism for flexible plant fronds^20^,^21^,^22^,^23^, and it should be interesting to conduct further research on the self-reconfiguration of the emergent aquatic macrophytes with a chiral morphology using a large-deformation theory of elastic beams.

### Relation between twisting chiral morphology and cross-sectional microstructure

As described above, twisting chiral morphology is a main feature of the emergent stalks/leaves of the aquatic macrophytes under investigation, except *Schoenoplectus tabernaemontani*. Besides their own weights, the twisted structures can be subjected to both transverse bending and axial torsion induced by wind. The optimal inner structures of the emergent stalks/leaves are well adapted to such loads. As shown in Fig. 2, all emergent stalks/leaves have porous cross sections with unique parenchyma foam microstructures, which not only allow air delivery and lighten the materials but also improve load-bearing capability. The parenchyma provides an array of strengthening ribs in the hollow stalk/leaf structures, which can be regarded as thin shells made of epidermis. According to the theory of elasticity^24^, the presence of inner parenchyma walls can greatly enhance the structural stability of the emergent stalks/leaves against torsional failure and buckling.

During the long history of natural evolution, the characteristic sizes and geometric structures of the inner parenchyma tissues may have been optimized together with the macroscopic configuration of the emergent stalks/leaves. This is consistent with our observations on aquatic macrophytes. In the emergent stalks of plants in Group I, which have a relatively small cross-sectional aspect ratio (*λ* ~ 1), the prism-packed parenchyma walls are connected into pentagonal or hexagonal chambers, somewhat like the structure of honeycombs. Honeycomb structures are widely used to enhance load-bearing capability without reduced quantity of material. The characteristic sizes of the chambers show a gradient variation in the radial direction, which further smoothens stress distribution. Therefore, the inner parenchyma structures in the emergent stalks may have been optimized together with the overall chiral morphology for maximal survivability. For the plants in Group II, e.g., *Acorus calamus* and *Typha orientalis*, which have a narrow cross section 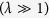, the slender emergent leaves have adopted a shape like the airplane wings. The inner parenchyma tissues are parallel to each other, dividing the leaf’s cross section into some straight-wall structures perpendicular to the epidermis. The parenchyma walls are approximately parallel to the more flexible direction of the leaf such that they can effectively improve the bending stiffness in this direction and prevent the leaf from buckling. Therefore, for both groups of aquatic macrophytes, both the macroscopic morphology and inner structures of the emergent stalks/leaves may have been adapted for mechanical stability. In spite of great variations in chiral morphologies and cross-sectional geometries, these plants appear to have all evolved with the objective to adapt to their natural environment.

## Conclusions

In summary, we have investigated, both experimentally and theoretically, the chiral growth tactics in several species of aquatic macrophytes. The chiral morphologies of their emergent stalks/leaves are found to be closely related to the large slenderness ratio, cross-sectional geometry, and loading conditions. Our analysis shows that the twisting chirality can significantly improve the resistance of the emergent stalks/leaves against failure under both internal and external loads. More specifically, for the aquatic macrophytes in Group I, which have head organs atop the emergent stalks, the main purpose of chiral growth seems to improve the load-bearing ability against Euler buckling, while for those in Group II without head organs, the chiral morphology can reduce their anisotropy in transverse bending resistance and the maximal stresses faced by them when subjected to wind. For most aquatic macrophytes with chiral morphologies, the twist angle per unit length of the emergent stalk/leaf increases with its cross-sectional aspect ratio. The chiral morphology, with the multiscale and functional gradient structure together, seems to have been optimized to endow the plants with superior mechanical properties to survive under varying environmental conditions. The present study has significantly deepened our understanding of the relations among morphology, mechanical property, biological functions and environmental loads of aquatic macrophytes.

Finally, it should be pointed out that real living systems need to strike a balance between mechanical properties and other physical/biological functions, and may not follow precisely the value of chirality that optimizes the mechanical properties alone. A distinct diversity of helical angles exists even for the same kind of plants. These aspects may also deserve further research.

## Methods

### Observations of cross sections

Fresh mature aquatic plants were collected as samples from July to September in Beijing, China. The intact and thick samples of emergent stalks/leaves were cut into segments by a sharp blade. Their cross sections were observed by using optical microscope (OM, Olympus IX71, China).

### Measurements of chiral configurations and cross-sectional geometry

For each plant, five samples of emergent stalks/leaves were selected for the measurement of chiral parameters. From the base to apex, each sample was cut into a few segments with a length of about 20 cm. For each segment, two terminal cross-sections and their relative axial rotation were recorded by digital camera (Canon IXUS 115 HS, Japan). The average twist angle per unit length, 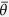, was determined from the total twist angle divided by the segment length. The cross-sectional aspect ratio, *λ*, was calculated from the average of its values at the two ends of the segment.

### Measurements of the mechanical properties of emergent stalks/leaves

Both quasi-static three-point bending tests and uniaxial tension tests of the emergent stalks/leaves were performed with a crosshead speed of 5.0 mm/min at the room temperature using universal testing machine (Zwick/Z005, Zwick/Roell Inc, Germany). Both the initial span in the bending tests and initial distance between clamps in the tension tests were set at 8 cm. For each plant, three emergent stalk/leaf samples were measured. From the base to apex, each sample was cut into a few segment of specimens with a length of about 15 cm. The force–displacement curves were recorded automatically. The cross-sections of the specimens were captured by a digital camera (Canon IXUS 115 HS, Japan).

### Theoretical model

The emergent stalk/leaf was modeled as a pre-twisted cantilever beam with a rectangular cross-section. For mathematical simplicity, our analysis has been restricted to linear kinematics, not accounting for the nonlinear geometric effects induced by large deflections, and we have neglected torsional buckling. For the slender plants under investigation, the influence of cross-sectional rotation on the bending behavior is relatively small and negligible. The Timoshenko beam model was adopted in our analysis. It was assumed that the beam is homogeneous, isotropic, linear elastic, and has a twist angle *θ* along its longitudinal direction. The governing equations were derived by using the principle of minimum potential energy. In the buckling analysis, the beam was subjected to a distributed compressive force (self-weight) *q* per unit length and a concentrated compressive load (head organs) *P* at the free end. Its critical buckling condition was determined using the finite element method. In the bending deformation and stress analysis, we assumed that the beam is subjected to a distributed transverse force (wind load), which is proportional to the upstream area and exerted on the beam in the undeformed configuration.

## Additional Information

**How to cite this article**: Zhao, Z.-L. *et al.* Biomechanical tactics of chiral growth in emergent aquatic macrophytes. *Sci. Rep.* **5**, 12610; doi: 10.1038/srep12610 (2015).

## Supplementary Material

Supplementary Information
