# Supplementary material for: Biomechanical tactics of chiral growth in emergent aquatic macrophytes
Source: Sci Rep. 2015 Jul 29;5:12610. doi: 10.1038/srep12610 (PMC4518234; doi:10.1038/srep12610)
Supplement: Supplementary Information [file srep12610-s1.pdf]

Supplementary Information for "Biomechanical tactics of chiral growth in emergent aquatic macrophytes"

Zi-Long Zhao, Hong-Ping Zhao, Bing-Wei Li, Ben-Dian Nie, Xi-Qiao Feng[*], and Huajian Gao

Detailed theoretical derivations

The detailed derivations pertaining to discussions in the main text on the effect of twisting chiral morphology on the mechanical properties of cantilever beams are given below.

We refer to the Cartesian coordinate system (x, y, z) shown in Fig. 6a, where the coordinate origin o is located at the cross-sectional centroid at the clamped end of the beam, x, y, and z axes are along its width b, thickness h, and length L directions, respectively. A twist coordinate system (X, Y, Z) is attached to the cross section of the beam, where the Z axis coincides with z, while X and Y axes rotate with the two principal directions of the cross-section.

We use Timoshenko beam theory with four generalized displacements, the deflections of the cross-sectional centroid, u_x and u_y, and the rotating angles of the cross section, φ_x and φ_y. The beam has a twist angle $\theta = \bar{\theta}L$ along its longitudinal direction, where $\bar{\theta}$ denotes the twist angle per unit length. The area moments of inertia I_{xx} and I_{yy}, and the polar moment of inertia I_{xy} are expressed as

$$I_{xx} = I_X \cos^2(\bar{\theta}z) + I_Y \sin^2(\bar{\theta}z),$$

$$I_{yy} = I_X \sin^2(\bar{\theta}z) + I_Y \cos^2(\bar{\theta}z),$$

$$I_{xy} = (I_Y - I_X)\sin(\bar{\theta}z)\cos(\bar{\theta}z), \qquad \text{(S.1)}$$

where $I_X = bh^3/12$ and $I_Y = b^3h/12$ denote the principal area moments of inertia of the cross section. Assume that the material is linear elastic and isotropic. The elastic strain energy of the beam due to bending and shearing is calculated as

$$U = \frac{1}{2}\int_0^L \left[EI_{xx}\left(\frac{\mathrm{d}\varphi_x}{\mathrm{d}z}\right)^2 + 2EI_{xy}\frac{\mathrm{d}\varphi_x}{\mathrm{d}z}\frac{\mathrm{d}\varphi_y}{\mathrm{d}z} + EI_{yy}\left(\frac{\mathrm{d}\varphi_y}{\mathrm{d}z}\right)^2 \right]\mathrm{d}z$$

$$+\frac{1}{2}\int_0^L \kappa GA\left[\left(\frac{\mathrm{d}u_x}{\mathrm{d}z} - \varphi_y\right)^2 + \left(\frac{\mathrm{d}u_y}{\mathrm{d}z} - \varphi_x\right)^2\right]\mathrm{d}z, \quad \text{(S.2)}$$

where E represents the Young's modulus, κ the shearing correction factor, $G = E/[2(1+v)]$ the shear modulus, v the Poisson's ratio, and A the cross-sectional area of the beam.

Euler buckling

We consider the Euler buckling of a pre-twisted beam with the clamp-free boundary condition and subjected to a distributed compressive force q and a concentrated compressive load P acting at the free end. The potential energy of the external forces is:

$$V = -\frac{qL}{2}\int_0^L \left(1-\frac{z}{L}\right)\left[\left(\frac{\mathrm{d}u_x}{\mathrm{d}z}\right)^2 + \left(\frac{\mathrm{d}u_y}{\mathrm{d}z}\right)^2\right]\mathrm{d}z - \frac{P}{2}\int_0^L \left[\left(\frac{\mathrm{d}u_x}{\mathrm{d}z}\right)^2 + \left(\frac{\mathrm{d}u_y}{\mathrm{d}z}\right)^2\right]\mathrm{d}z. \quad \text{(S.3)}$$

The principle of minimum potential energy requires that

$$\delta\Pi = \delta(U+V) = 0. \quad \text{(S.4)}$$

The boundary conditions are

$$u_x|_{z=0} = u_y|_{z=0} = \varphi_x|_{z=0} = \varphi_y|_{z=0} = 0. \quad \text{(S.5)}$$

The critical condition of the pre-twisted beam can be solved from Eqs. (S.4) and (S.5) by using the finite element method (1).

Bending

We further investigate the bending property of the pre-twisted cantilever beam subjected to distributed transverse force. Assume that the force acting on the beam is proportional to the upstream area. The transverse force f_y with intensity $\bar{f}$ per unit area has the form of

$$f_y = f_1 + f_2\left|\cos(\bar{\theta}z)\right|, \quad 0 \le z \le L, \tag{S.6}$$

where $f_1 = h\bar{f}$ and $f_2 = (b-h)\bar{f}$.

When $0° \le \theta \le 90°$, one can derive the static equilibrium equations

$$\begin{aligned}
&\frac{\mathrm{d}^2u_x}{\mathrm{d}z^2} = \frac{\mathrm{d}\varphi_y}{\mathrm{d}z},\\
&\frac{\mathrm{d}^2u_y}{\mathrm{d}z^2} = -\frac{f_1 + f_2\cos(\bar{\theta}z)}{\kappa GA} + \frac{\mathrm{d}\varphi_x}{\mathrm{d}z},\\
&\frac{\mathrm{d}}{\mathrm{d}z}\left(I_{xx}\frac{\mathrm{d}\varphi_x}{\mathrm{d}z} + I_{xy}\frac{\mathrm{d}\varphi_y}{\mathrm{d}z}\right) = \frac{\kappa GA}{E}\left(\varphi_x - \frac{\mathrm{d}u_y}{\mathrm{d}z}\right),\\
&\frac{\mathrm{d}}{\mathrm{d}z}\left(I_{xy}\frac{\mathrm{d}\varphi_x}{\mathrm{d}z} + I_{yy}\frac{\mathrm{d}\varphi_y}{\mathrm{d}z}\right) = \frac{\kappa GA}{E}\left(\varphi_y - \frac{\mathrm{d}u_x}{\mathrm{d}z}\right),
\end{aligned} \tag{S.7}$$

and boundary conditions

$$\begin{aligned}
&u_x|_{z=0} = u_y|_{z=0} = \varphi_x|_{z=0} = \varphi_y|_{z=0} = 0,\\
&\kappa GA\left(\frac{\mathrm{d}u_x}{\mathrm{d}z} - \varphi_y\right)|_{z=L} = \kappa GA\left(\frac{\mathrm{d}u_y}{\mathrm{d}z} - \varphi_x\right)|_{z=L}\\
&\quad = \left(EI_{xx}\frac{\mathrm{d}\varphi_x}{\mathrm{d}z} + EI_{xy}\frac{\mathrm{d}\varphi_y}{\mathrm{d}z}\right)|_{z=L} = \left(EI_{yy}\frac{\mathrm{d}\varphi_y}{\mathrm{d}z} + EI_{xy}\frac{\mathrm{d}\varphi_x}{\mathrm{d}z}\right)|_{z=L} = 0,
\end{aligned} \tag{S.8}$$

of the beam. From Eqs. (S.7) and (S.8), u_x and u_y can be derived as (1)

$$\begin{aligned}
&u_x = \frac{I_X - I_Y}{144\bar{\theta}^4EI_XI_Y}(9f_1C_{1x} + 2f_2C_{2x}),\\
&u_y = \frac{1}{288\bar{\theta}^4EI_Y}\left(3f_1C_{1y} + f_2C_{2y}\right),
\end{aligned} \tag{S.9}$$

where

$$\begin{aligned}
C_{1x} &= \left[\frac{3}{2} - (\theta-\alpha)^2\right]\sin2\alpha + 2(\theta-\alpha)\cos2\alpha - 2\theta + (2\theta^2-1)\alpha,\\
C_{2x} &= 2[10 + 2\cos2\alpha - 9\cos(\theta-\alpha) - 9(\theta-\alpha)\sin\theta\cos\alpha]\sin\alpha\\
&\quad +18\alpha\left(\cos\theta + \theta\sin\theta - \frac{4}{3}\right),\\
C_{1y} &= 48\beta\theta^2\alpha(2\theta-\alpha) + 2\alpha^2(1+\lambda)(6\theta^2 - 4\theta\alpha + \alpha^2)\\
&\quad +3(1-\lambda)\{[2(\theta-\alpha)^2 - 3]\cos2\alpha + 4(\theta-\alpha)\sin2\alpha + 3 - 2\theta^2 - 4\theta\alpha\},\\
C_{2y} &= 288[(1+\beta\theta^2)(\cos\alpha - 1) + \beta\theta^2\alpha\sin\theta]\\
&\quad +24\alpha^2(1+\lambda)(3\cot\theta + 3\theta - \alpha)\sin\theta\\
&\quad +4(1-\lambda)\{56 - 54\cos\alpha - 2\cos3\alpha\\
&\qquad +9[\sin2\alpha - \alpha\cos2\alpha - \alpha - 2(\cot\theta + \theta)\sin^2\alpha]\sin\theta\},
\end{aligned}$$

$$\alpha = \bar{\theta} z,$$

$$\beta = \frac{EI_Y}{\kappa GAL^2}. \tag{S.10}$$

When $\theta > 90°$, the transverse force f_y can be expressed by using the Fourier series expansion. The deflections of the beam can be determined by resorting to the superposition principle.

The normal strain at the position (x, y, z) is written as

$$\varepsilon_z = x\frac{\mathrm{d}\varphi_y}{\mathrm{d}z} + y\frac{\mathrm{d}\varphi_x}{\mathrm{d}z}. \tag{S.11}$$

When $0° \le \theta \le 90°$, we have

$$\frac{\mathrm{d}\varphi_y}{\mathrm{d}z} = \frac{\mathrm{d}^2 u_x}{\mathrm{d}z^2},$$

$$\frac{\mathrm{d}\varphi_x}{\mathrm{d}z} = \frac{\mathrm{d}^2 u_y}{\mathrm{d}z^2} + \frac{f_1 + f_2\cos(\bar{\theta} z)}{\kappa GA}. \tag{S.12}$$

The normal stress at the position (x, y, z) is then written as

$$\sigma_z = Ex\frac{\mathrm{d}^2 u_x}{\mathrm{d}z^2} + Ey\frac{\mathrm{d}^2 u_y}{\mathrm{d}z^2} + Ey\frac{f_1 + f_2\cos\alpha}{\kappa GA}. \tag{S.13}$$

For each cross section, the maximum normal stress always happens at one of its corners. The maximum normal stress is

$$\sigma_z^* = \max\left\{E\left|\frac{b\cos\alpha \mp h\sin\alpha}{2}\frac{\mathrm{d}^2 u_x}{\mathrm{d}z^2}\right| + E\left|\frac{b\sin\alpha \pm h\cos\alpha}{2}\left(\frac{\mathrm{d}^2 u_y}{\mathrm{d}z^2} + \frac{f_1 + f_2\cos\alpha}{\kappa GA}\right)\right|\right\}. \tag{S.14}$$

Substituting Eq. (S.9) into (S.14) yields

$$\sigma_z^* = \max\left\{\left|E(b\cos\alpha \mp h\sin\alpha)\frac{(I_X - I_Y)\left(9f_1 C_{1x,zz} + 2f_2 C_{2x,zz}\right)}{288\bar{\theta}^4 EI_X I_Y}\right| + \left|E(b\sin\alpha \pm h\cos\alpha)\left(\frac{3f_1 C_{1y,zz} + f_2 C_{2y,zz}}{576\bar{\theta}^4 EI_Y} + \frac{f_1 + f_2\cos\alpha}{2\kappa GA}\right)\right|\right\}. \tag{S.15}$$

where

$$C_{1x,zz} = 4\bar{\theta}^2(\theta - \alpha)^2\sin 2\alpha,$$

$$C_{2x,zz} = 36\bar{\theta}^2[\cos\theta - \cos\alpha + (\theta - \alpha)\sin\theta]\sin 2\alpha,$$

$$C_{1y,zz} = 24\bar{\theta}^2\{(\theta - \alpha)^2[(1 + \lambda) - (1 - \lambda)\cos 2\alpha] - 4\beta\theta^2\},$$

$$C_{2y,zz} = 72\bar{\theta}^2\{-4(1 + \beta\theta^2)\cos\alpha + 2(1 + \lambda)(\theta - \alpha + \cot\theta)\sin\theta + (1 - \lambda)[3\cos\alpha + \cos 3\alpha - 2\cos 2\alpha(\cos\theta + \theta\sin\theta - \alpha\sin\theta)]\}.$$

(S.16)

Let γ denote the direction of $\boldsymbol{f}(z)$ measured clockwise from the y direction. When $\gamma = 0°$ and $b \geq h$, the normal stress in the beam reaches the maximum at the clamped end ($z = 0$). Letting $z = 0$, the maximum normal stress in the beam can be derived as

$$\sigma_{\max} = \sigma_z^*|_{z=0} = \frac{hL^2}{4I_X}\left|f_1 + 2f_2\frac{\cos\theta + \theta\sin\theta - 1}{\theta^2}\right|. \tag{S.17}$$

As the twist angle $\theta \to 0$, we have

$$\sigma_{\max}^{(0)} = \lim_{\theta\to 0}\sigma_{\max} = \frac{hL^2}{4I_X}(f_1 + f_2). \tag{S.18}$$

For an untwisted beam (i.e., $\theta = 0$), the result in Eq. (S.18) reduces to the classical solution, where $f_1 + f_2$ is the distributed transverse force exerted on the beam (2).

Normalize the maximum normal stress as

$$\bar{\sigma}_{\max} = \frac{\sigma_{\max}}{\sigma_{\max}^{(0)}} = \left|\frac{h}{b} + 2\left(1 - \frac{h}{b}\right)\frac{\cos\theta + \theta\sin\theta - 1}{\theta^2}\right|. \tag{S.19}$$

For a beam with a square cross section (i.e., $\lambda = 1$), we have $\bar{\sigma}_{\max} \equiv 1$, meaning that pre-twisting has no effect on its maximum normal stress.

When $\theta > 90°$, the transverse force f_y can be expanded as

$$f_y = f_1 + f_2|\cos(\bar{\theta}z)| = f_1 + f_2a_0 + f_2\sum_{n=1}^{\infty}a_n\cos\left(\frac{n\pi z}{L}\right), \tag{S.20}$$

where the coefficients a_0 and a_n are

$$a_0 = \frac{1}{L}\int_0^L|\cos(\bar{\theta}z)|\mathrm{d}z = \int_0^1|\cos(\theta\bar{z})|\mathrm{d}\bar{z},$$

$$a_n = \frac{2}{L}\int_0^L|\cos(\bar{\theta}z)|\cos\left(\frac{n\pi z}{L}\right)\mathrm{d}z = 2\int_0^1|\cos(\theta\bar{z})|\cos(n\pi\bar{z})\mathrm{d}\bar{z}, \tag{S.21}$$

with $\bar{z} = z/L$.

Then for the case of $\theta > 90°$, the normalized normal stress $\bar{\sigma}_{\max}$ can be derived by using the superposition principle.

On optimization metric for chiral growth

To better elucidate the chiral growth tactics of aquatic macrophytes, reasonable mechanical metrics should be employed in the theoretical analyses according to the loading conditions. For example, the maximum stress encountered by the plant can be directly related to failure, thus could serve as an essential metric in the bending analysis. Generally, the chiral morphology of these slender plants might be related to a multitude of mechanical functions, e.g., reducing the maximum stress, promoting reconfiguration, and improving the resistance against dislodging and axial buckling. These mechanisms are most likely not standing-alone but work synergistically to help the plants adapt to different environmental conditions.

Besides, the large amplitude effects (e.g., wind-induced torsion, wind-adaptive reconfiguration, and flutter instabilities) of the plant fronds when subjected to wind are also of interest and deserve further study. Fluid dynamics simulations and wind tunnel experiments can be carried out to further reveal these aerodynamic mechanisms. We have performed some preliminary fluid dynamics simulations by using the immersed boundary method. The results show that the simplifications made in the present theoretical analysis do not interfere with the main conclusions drawn from this study. The effects of fluid-structure interaction will be investigated in details in the next step.

References

1. Zhao, Z.L., Zhao, H.P., Chang, Z., Feng, X.Q. Analysis of bending and buckling of pre-twisted beams: A bioinspired study. *Acta Mech. Sin.* **30**, 507–515 (2014).
2. Timoshenko, S.P., Goodier J.N. Theory of Elasticity, 3rd Edition (McGraw-Hill, New York, 1969).
